# Remote platelet function testing using P-selectin expression in patients with recent cerebral ischaemia on clopidogrel

**DOI:** 10.1136/svn-2020-000346

**Published:** 2020-09-24

**Authors:** Jason Philip Appleton, Carla Richardson, Natalia Dovlatova, Jane May, Nikola Sprigg, Stan Heptinstall, Philip M Bath

**Affiliations:** 1 Stroke Trials Unit, Division of Clinical Neuroscience, University of Nottingham, Nottingham, UK; 2 Stroke Division, Nottingham University Hospitals NHS Trust, Nottingham, UK; 3 Platelet Solutions Ltd, Division of Clinical Neuroscience, Queen's Medical Centre, University of Nottingham, Nottingham, UK; 4 Platelet Research Group, Division of Clinical Neuroscience, Queen's Medical Centre, University of Nottingham, Nottingham, UK

**Keywords:** stroke

## Abstract

**Background:**

Antiplatelet agents reduce recurrence after cerebral ischaemia but are not effective in all patients, in part because of treatment resistance. The primary aim was to assess the proportion of patients who are insensitive to clopidogrel. The secondary aim was to assess the association between insensitivity to clopidogrel and recurrent cerebrovascular events.

**Methods:**

Following written informed consent, independent patients with a recent non-cardioembolic ischaemic stroke or transient ischaemic attack, and taking clopidogrel, were enrolled. Platelet function was assessed with remote measurement of surface expression of P-selectin (CD62P) using commercial kits sensitive to aspirin or clopidogrel. Participants’ general practitioners provided details on recurrent vascular events at least 90 days later. Data are mean (SD) and median [IQR]. Resistance was defined as: aspirin median fluorescence (MF) >500 units, clopidogrel MF >860 units. Non-parametric descriptors and tests were used.

**Results:**

63 patients were recruited: mean age 64 (13.7) years, women 47%. At baseline, 59 (95%) patients were taking clopidogrel alone with 3 (5%) on combined clopidogrel and aspirin. Assessment of platelet surface P-selectin revealed: aspirin test 528 [317, 834], >500 54.8%; clopidogrel test 429 [303, 656], >860 11.3%. No participants on aspirin and clopidogrel showed aspirin resistance. Thirteen (20.6%) patients had a recurrent cerebrovascular event; those with an ischaemic stroke had a non-significantly higher baseline P-selectin using the clopidogrel test as compared with those with no recurrence: 626 [380, 801] versus 406 [265, 609], p=0.08.

**Conclusions:**

Remote measurement of platelet function assessed using the platelet surface expression of P-selectin is feasible. 11% of patients taking clopidogrel showed resistance. No significant associations were noted between clopidogrel resistance and recurrent ischaemic events.

## Introduction

Antiplatelet drugs are effective at reducing recurrent events after ischaemic stroke or transient ischaemic attack (TIA).^1–6^ Unlike other secondary prevention interventions such as blood pressure and lipid lowering, antithrombotic antiplatelet agents are prescribed and administered without ongoing monitoring as there are no widely available, affordable, effective and validated tests of platelet function. Despite this, over a third of patients in some cohorts are insensitive to the antiplatelet effects of clopidogrel^7 8^; a smaller proportion of patients appear to be resistant to aspirin.^8 9^

Surface platelet expression of P-selectin (CD62P) correlates with other platelet function measures and can be tested remotely from the patient,^10^ in contrast to other techniques that cannot be used remotely.^11 12^ Antiplatelet resistance has been defined for aspirin as median fluorescence (MF) >500 units, and for clopidogrel MF >860 units in separate studies assessing acute coronary syndrome,^13^ and ischaemic stroke and patients with TIA, respectively.^14^ In a substudy of the large Triple Antiplatelets for Reducing Dependency after Ischaemic Stroke (TARDIS) trial, remote assessment of P-selectin was feasible and found that aspirin and clopidogrel reduced stimulated P-selectin although 25% of patients on clopidogrel had high on-treatment platelet activity.^14^

We assessed the proportion of patients with a history of cerebral ischaemia who were insensitive to clopidogrel and the association with recurrent ischaemic or bleeding events. Our primary aim was to assess the proportion of patients who are insensitive to clopidogrel. The secondary aim was to assess the association between insensitivity to clopidogrel and recurrent cerebrovascular events.

## Methods

### Population

Independent adults (modified Rankin Scale, mRS≤3) aged ≥40 years at high risk of recurrent ischaemic stroke or TIA were eligible for recruitment if they had a clinical diagnosis of ischaemic non-cardioembolic stroke or TIA, were on clopidogrel for at least 5 days prior to recruitment and were able to provide written informed consent. Patients on anticoagulation (eg, warfarin, direct oral anticoagulants, heparin) were excluded. Patients were recruited from the stroke service (inpatient or outpatient) at Nottingham University Hospitals NHS Trust. Participants were asked to complete a short questionnaire and underwent a blood test on the day of recruitment. We aimed to recruit 60 patients to assess the proportion of patients resistant to clopidogrel. The study was not powered to assess the association between P-selectin clopidogrel test values and clinical outcomes. The investigator-initiated study was funded by Platelet Solutions (Nottingham, UK) and Innovate UK SMART award, and sponsored by the University of Nottingham.

### P-selectin assay

P-selectin is derived from alpha-granules within platelets and becomes exposed on the platelet surface membrane when platelets are activated. Measurement of surface expression of P-selectin was performed using commercial kits sensitive to aspirin or clopidogrel (Platelet Solutions). Citrate anticoagulated blood was kept at 37°C once collected using a dry heat pad and insulation pouch, then incubated with platelet stimulants: arachidonic acid 0.5 mM for aspirin testing; ADP 10 µM for clopidogrel testing; and an unstimulated sample for baseline expression. After 5 min of incubation, a fixative (PAMFix)^15^ was added and the fixed samples were transferred to the Nottingham flow cytometry laboratory for processing. Fixed blood was incubated with fluorescent antibodies to identify platelets (CD61) and P-selectin (CD62P). MF was recorded for platelet surface expression of P-selectin for each sample.

### Clinical outcomes

A questionnaire was completed by participants on recruitment detailing any history of bleeding while on antiplatelet medication. Participants’ general practitioners (GP) were contacted at least 90 days later to record any recurrent vascular events and confirmed with admitting hospital records where available. An additional follow-up telephone call at least 90 days following recruitment was performed in a subset of patients who provided separate written informed consent. Clinical outcomes were recorded, including: functional status (mRS); disability (Barthel Index); quality of life (European Quality of Life Visual Analogue Scale); cognition (telephone Mini-Mental State Examination), Modified Telephone Interview for Cognition Scale, verbal fluency); and mood (Zung Depression Scale).

### Statistics

Resistance was defined prospectively using cut-offs previously utilised in stroke patients in a large randomised controlled trial: aspirin MF >500 units, clopidogrel MF >860 units.^14^ Data are number (%), mean (SD) and median [IQR]. Baseline differences in groups based on antiplatelet therapy were assessed using analysis of variance for continuous variables or χ^2^ for categorical variables. Non-parametric descriptors and tests were used with significance set at p<0.05. Correlations between baseline P-selectin clopidogrel test values and clinical outcomes were assessed using Spearman correlation with resultant ‘r’ and 2-sided p values. Analyses were performed using SPSS V.24.

### Data availability

Data pertaining to this paper are available from the corresponding author on reasonable request.

## Results

Sixty-three patients were recruited although one had no data or blood collected. Of the remaining 62 participants, 53% were men with an average age of 64 years (table 1). The index event was stroke in 44 (71%) participants and TIA in the remaining 29%. All participants were on clopidogrel 75 mg daily and three were also on aspirin 75 mg daily at the time of recruitment. Participants on dual antiplatelets tended to have a prior history of TIA reflecting the results of the Clopidogrel in High-Risk Patients with Acute Nondisabling Cerebrovascular Events (CHANCE) trial.^4^ Time from index event to recruitment was longer in those on clopidogrel only versus aspirin and clopidogrel. Seventeen (27%) participants had a history of minor bleeding on clopidogrel, with the remainder reporting no history of bleeding (table 1).

**Table 1:** Baseline characteristics of participants

	All	Clopidogrel only	Aspirin+clopidogrel	P value
n (%)	62 (100.0)	59 (95.2)	3 (4.8)	
Male (%)	33 (53.2)	33 (55.9)	0	0.058
Age, years	63.7 (13.7)	63.0 (13.5)	78.0 (8.9)	0.06
Preindex event				
Stroke (%)	9 (14.5)	8 (13.6)	1 (33.3)	0.34
TIA (%)	8 (12.9)	6 (10.2)	2 (66.7)	0.004
IHD (%)	6 (9.7)	5 (8.5)	1 (33.3)	0.16
Diabetes (%)	11 (17.7)	10 (16.9)	1 (33.3)	0.47
Smoking (%)	9 (14.5)	9 (15.3)	0	0.74
Index event				
Ischaemic stroke (%)	44 (71.0)	42 (71.2)	2 (66.7)	0.87
TIA (%)	18 (29.0)	17 (28.8)	1 (33.3)	0.87
Thrombolysis (%)	4 (6.5)	4 (6.8)	0	0.64
Length of stay, days	1 [1,3] (0–60)	1 [0,3] (0–60)	2 [−] (1-7)	0.25
At recruitment				
Time from index event, days	49 [35,75] (1–285)	52 [36,76] (1–285)	1 [−] (1–22)	0.006
mRS	0 [0,1]	0 [0,1]	0 [−]	0.89
On clopidogrel (%)	62 (100)	59 (100)	3 (100)	–
Time on clopidogrel, days	50 [35, 76] (8–1878)	48 [35, 69] (8–1878)	313 [−] (8–962)	0.41
On aspirin (%)	3 (4.8)	0	3 (100)	–
Treated hypertension (%)	43 (69.4)	40 (67.8)	3 (100)	0.24
Treated hypercholesterolaemia (%)	59 (95.2)	56 (94.9)	3 (100)	0.69
Proton pump inhibitor (%)	19 (30.6)	17 (28.8)	2 (66.7)	0.17
Systolic BP, mm Hg	135.7 (19.5)	134.9 (19.3)	150.0 (22.5)	0.21
Diastolic BP, mm Hg	76.3 (13.5)	76.6 (13.7)	71.7 (7.8)	0.26
Bleeding history on antiplatelet(s)				
Minor bleeding (%)	17 (27.4)	17 (28.8)	0	0.28
Major bleeding (%)	0	0	0	–

Data are number (%), mean (SD), median [IQR], (min–max); comparison between clopidogrel versus aspirin+clopidogrel groups by χ^2^ test, Kruskal-Wallis test or one-way analysis of variance.

Sixty-three participants recruited but 1 had no data collected.

BP, blood pressure; IHD, ischaemic heart disease; mRS, modified Rankin Scale; TIA, transient ischaemic attack.

**Figure 1:**
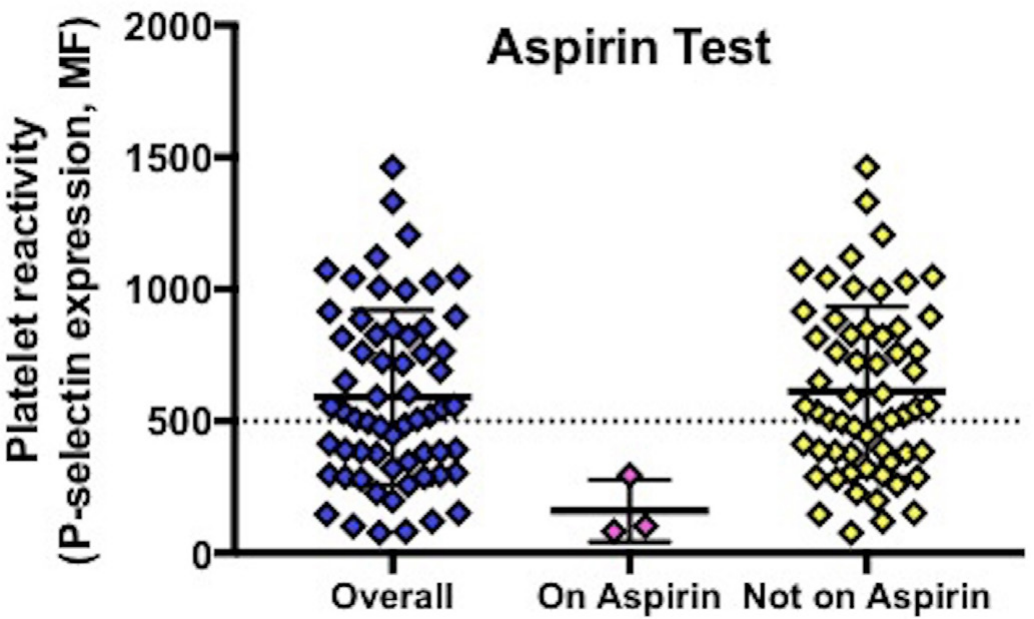
P-selectin surface expression in response to arachidonic acid. MF, median fluorescence.

**Figure 2:**
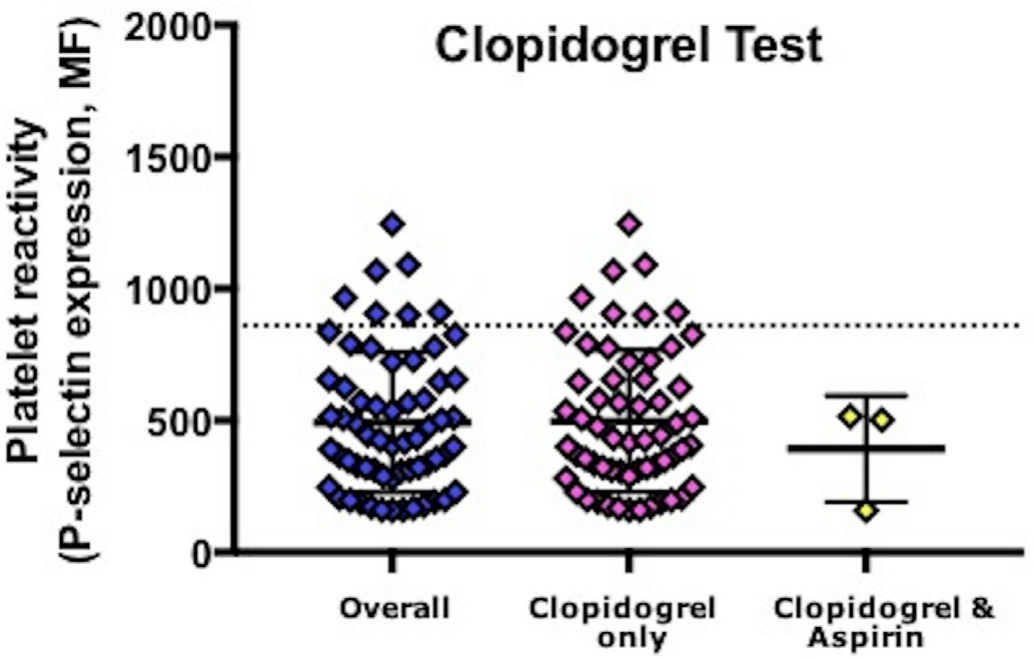
P-selectin surface expression in response to ADP. MF, median fluorescence.

Assessment of platelet surface P-selectin in 62 participants revealed (table 2): aspirin test 528 [317, 834], >500 54.8%, figure 1; clopidogrel test 429 [303, 656], >860 11.3%, figure 2. Thus, 11.3% of participants had evidence of resistance to clopidogrel. No participants on aspirin and clopidogrel showed aspirin resistance. P-selectin levels for the clopidogrel test did not differ between patients taking and not taking a proton pump inhibitor (PPI 554 [307, 729] vs no PPI 406 [289, 626] (p=0.32)) or between patients with and without a history of bleeding (aspirin test: bleeding 691 [438,791] vs no bleeding 495 [296, 870], p=0.28; clopidogrel test: 444 [321, 685] vs 425 [285, 656], p=0.88) (table 3). No patients with minor bleeding were resistant to clopidogrel (clopidogrel test >860), while 7 (15.6%) patients with no bleeding symptoms were resistant to clopidogrel.

**Table 2:** P-selectin in response to arachidonic acid (AA, aspirin test) and ADP (clopidogrel test)

	All	Clopidogrel only	Aspirin +clopidogrel	P value
AA MF				
Number (%)	62 (100)	59 (95.2)	3 (4.8)	–
Mean (SD)	590 (330)	612 (323)	160 (118)	0.020
Median [IQR]	528 [317, 834]	547 [375,851]	103 [−]	0.013
>500 (%)	34 (54.8)	34 (57.6)	0	0.050
ADP MF				
Number (%)	62 (100)	59 (95.2)	3 (4.8)	–
Mean (SD)	493 (266)	498 (269)	392 (202)	0.51
Median [IQR]	429 [303, 656]	425 [307, 656]	502 [−]	0.54
>860 (%)	7 (11.3)	7 (11.9)	0	0.53

Data are number (%), median [IQR] or mean (SD); comparisons by χ^2^ test, Kruskal-Wallis test or one-way analysis of variance.

MF, median fluorescence.

**Table 3:** P-selectin in response to arachidonic acid (AA, aspirin test) and ADP (clopidogrel test) by minor or no bleeding

	All	Minor bleeding	No bleeding	P value
AA MF				
Number (%)	62 (100)	17 (27.4)	45 (72.6)	–
Mean (SD)	590 (330)	644 (280)	569 (348)	0.43
Median [IQR]	528 [317, 834]	691 [438, 791]	495 [296, 870]	0.28
>500 (%)	34 (54.8)	12 (70.6)	22 (48.9)	0.13
ADP MF				
Number (%)	62 (100)	17 (27.4)	45 (72.6)	–
Mean (SD)	493 (266)	481 (213)	498 (286)	0.83
Median [IQR]	429 [303, 656]	444 [321, 685]	425 [285, 656]	0.88
>860 (%)	7 (11.3)	0	7 (15.6)	0.08

Data are number (%), median [IQR] or mean (SD); comparisons by χ^2^ test, Kruskal-Wallis test or one-way analysis of variance.

MF, median fluorescence.

GP follow-up data were available for all participants. Thirteen (20.6%) participants had a recurrent vascular event during the follow-up period: nine ischaemic strokes and four TIAs. No participants died or had acute coronary syndromes during the follow-up period. Baseline P-selectin clopidogrel test levels were non-significantly higher in those participants that had a recurrent ischaemic stroke as compared with those without recurrence (626 [380, 801] vs 406 [265, 609], p=0.08). Only one participant with recurrent ischaemic stroke had baseline P-selectin clopidogrel test >860 suggestive of clopidogrel resistance. When pooled together, those with either a recurrent ischaemic stroke or TIA had non-significantly higher baseline P-selectin clopidogrel test levels compared with those with no recurrence (581 [328, 752] vs 406 [239, 609], p=0.15). Only 1/13 (7.7%) participant with recurrent ischaemic stroke or TIA had a baseline P-selectin clopidogrel test >860 as compared with 6/49 (12.2%) participants with no recurrence (p=0.65). There was no statistically significant correlation between baseline P-selectin clopidogrel test levels and recurrent cerebrovascular events using an ordinal scale where no event=0, TIA=1 and ischaemic stroke=2 (r=0.200, p=0.12).

Telephone follow-up data were only available for 19 (30.2%) participants. No statistically significant correlations were noted between baseline P-selectin clopidogrel test levels and clinical outcomes (table 4).

**Table 4:** Correlations between baseline P-selectin clopidogrel test and clinical outcomes after day 90

	r	P value
Number (%)	19 (30.2)	–
mRS	0.164	0.50
Barthel index	−0.208	0.39
EQ-VAS	0.075	0.76
t-MMSE	−0.060	0.81
TICS-M	−0.231	0.34
Verbal fluency	−0.235	0.33
Zung	−0.125	0.61

Data are number (%), r with two-sided p values using Spearman correlation.

EQ-VAS, European Quality of Life Visual Analogue Scale; mRS, modified Rankin Scale; TICS-M, Modified Telephone Interview for Cognition Scale; t-MMSE, telephone Mini-Mental State Examination.

## Discussion

In this single centre study, remote measurement of platelet function assessed using the platelet surface expression of P-selectin was feasible. Over a tenth of patients taking clopidogrel showed resistance. There were non-significantly higher baseline P-selectin clopidogrel test levels in those participants who had a recurrent cerebral ischaemic event as compared with those with no recurrence.

The proportion of patients demonstrated to be resistant to clopidogrel varies in the literature but may exceed 35%.^16^ A variety of platelet function tests have been reported, but the majority need to be performed in a timely manner close to the patient. In comparison, platelet surface expression of P-selectin provides unique remote assessment of platelet function distal to the patient and has been shown to be feasible in healthy volunteers as well as patients with acute coronary syndrome, surgery and mild bleeding disorders.^16^ Furthermore, storage and transportation of the fixed samples to a core laboratory for analysis were feasible across multiple hospital sites as part of a large clinical trial of stroke and TIA patients.^14^ This analysis demonstrated that 24.7% of 97 patients on clopidogrel at baseline demonstrated clopidogrel resistance defined as MF >860,^14^ a higher proportion than in the present study.

In patients with acute coronary syndrome, clopidogrel resistance (demonstrated using platelet surface P-selection expression) has been shown to be associated with an increased risk of cardiovascular death, acute coronary syndrome or stent thrombosis.^13^ The present study is the first to assess the association between baseline platelet surface expression of P-selectin indicative of clopidogrel resistance in patients with prior ischaemic stroke or TIA and recurrent cerebrovascular events. Although there was no association when using predefined cut points, median P-selectin clopidogrel test values were non-significantly higher in those with a recurrent vascular event than in those without. This finding needs to be replicated in larger datasets, but is in line with another study using thromboelastography ADP maximum amplitude as a marker of high on-treatment platelet reactivity, which was associated with a higher risk of recurrent ischaemic events at 90 days.^17^ However, there is an absence of trials assessing whether adjusting antiplatelet therapy post stroke in patients with clopidogrel resistance is feasible, safe and efficacious at reducing recurrent cerebrovascular events. Such adjustment might comprise switching from clopidogrel to aspirin, using combined aspirin and clopidogrel,^4 5^ or using combined aspirin and dipyridamole.^6 18 19^ In this respect, it is interesting to note in the present study that all patients on combined aspirin and clopidogrel had suppressed P-selectin expression with both of the aspirin and clopidogrel tests.

The strength of this study is its generalisability to clinical practice and further demonstration that platelet function can be assessed remotely after stroke, in this case in a predominantly outpatient population. However, there are several limitations. First, the study was small and this limited statistical power to assess the relationship between P-selectin expression and clinical outcome. This is particularly relevant for the binary comparison of non-supressed P-selectin versus stroke recurrence. Further, participants were assessed around 7 weeks after their index event and so at a time when the risk of recurrence was likely to be low. Second, as a predominantly outpatient population, the participants tended to have a mild stroke or TIA and so the results cannot be extrapolated to a more severe population. Third, we did not collect the time when antiplatelet medication was last taken and so were unable to assess whether the time from medication taken to blood testing impacting on the results. Last, we cannot rule out that some participants were non-compliant with their antiplatelet therapy.

In summary, we add to the growing body of evidence that remote assessment of platelet function using P-selectin is feasible and of potential benefit to patient management. Just over a tenth of patients were resistant to clopidogrel. Although there was a non-significant association with recurrent cerebrovascular events in those resistant to clopidogrel, the study was not powered to answer this definitively. Therefore, further studies are needed to establish whether platelet function testing can be used to guide antiplatelet therapy in patients who appear to be resistant to clopidogrel.

## References

[1] Antithrombotic Trialists' Collaboration. Collaborative meta-analysis of randomised trials of antiplatelet therapy for prevention of death, myocardial infarction, and stroke in high risk patients. BMJ 2002;324:71–86. 10.1136/bmj.324.7329.71 11786451 PMC64503

[2] CAPRIE Steering Committee. A randomised, blinded, trial of clopidogrel versus aspirin in patients at risk of ischaemic events (CAPRIE). CAPRIE Steering Committee. Lancet 1996;348:1329–39. 10.1016/S0140-6736(96)09457-3 8918275

[3] Halkes PHA , Gray LJ , Bath PMW , et al. Dipyridamole plus aspirin versus aspirin alone in secondary prevention after TIA or stroke: a meta-analysis by risk. J Neurol Neurosurg Psychiatry 2008;79:1218–23. 10.1136/jnnp.2008.143875 18535024

[4] Wang Y , Wang Y , Zhao X , et al. Clopidogrel with aspirin in acute minor stroke or transient ischemic attack. N Engl J Med 2013;369:11–19. 10.1056/NEJMoa1215340 23803136

[5] Johnston SC , Easton JD , Farrant M , et al. Clopidogrel and aspirin in acute ischemic stroke and high-risk TIA. N Engl J Med 2018;379:215–25. 10.1056/NEJMoa1800410 29766750 PMC6193486

[6] Sacco RL , Diener H-C , Yusuf S , et al. Aspirin and extended-release dipyridamole versus clopidogrel for recurrent stroke. N Engl J Med 2008;359:1238–51. 10.1056/NEJMoa0805002 18753638 PMC2714259

[7] Gurbel PA , Bliden KP , Hiatt BL , et al. Clopidogrel for coronary stenting: response variability, drug resistance, and the effect of pretreatment platelet reactivity. Circulation 2003;107:2908–13. 10.1161/01.CIR.0000072771.11429.83 12796140

[8] Fontana P , Nolli S , Reber G , et al. Biological effects of aspirin and clopidogrel in a randomized cross-over study in 96 healthy volunteers. J Thromb Haemost 2006;4:813–9. 10.1111/j.1538-7836.2006.01867.x 16634751

[9] Hankey GJ , Eikelboom JW . Aspirin resistance. Lancet 2006;367:606–17. 10.1016/S0140-6736(06)68040-9 16488805

[10] Fox SC , May JA , Shah A , et al. Measurement of platelet P-selectin for remote testing of platelet function during treatment with clopidogrel and/or aspirin. Platelets 2009;20:250–9. 10.1080/09537100902912451 19440925

[11] Harrison P , Segal H , Blasbery K , et al. Screening for aspirin responsiveness after transient ischemic attack and stroke: comparison of 2 point-of-care platelet function tests with optical aggregometry. Stroke 2005;36:1001–5. 10.1161/01.STR.0000162719.11058.bd 15817896

[12] Harrison P , Segal H , Silver L , et al. Lack of reproducibility of assessment of aspirin responsiveness by optical aggregometry and two platelet function tests. Platelets 2008;19:119–24. 10.1080/09537100701771736 18297549

[13] Thomas MR , Wijeyeratne YD , May JA , et al. A platelet P-selectin test predicts adverse cardiovascular events in patients with acute coronary syndromes treated with aspirin and clopidogrel. Platelets 2014;25:612–8. 10.3109/09537104.2013.863858 24433232

[14] Bath PM , May J , Flaherty K , et al. Remote assessment of platelet function in patients with acute stroke or transient ischaemic attack. Stroke Res Treat 2017;2017:1–13. 10.1155/2017/7365684 PMC5463170 28630782

[15] Heptinstall S , Fox S , May J , et al. PAMFix, a fixative developed to enable remote platelet function testing: uses and applications. Current Topics in Pharmacology 2015;19:1–12.

[16] Bath PM , May J , Heptinstall S . Clinical utility of remote platelet function measurement using P-selectin: assessment of aspirin, clopidogrel, and prasugrel and bleeding disorders. Platelets 2018;29:425–30. 10.1080/09537104.2018.1445839 29667460

[17] Rao Z , Zheng H , Wang F , et al. High on-treatment platelet reactivity to adenosine diphosphate predicts ischemic events of minor stroke and transient ischemic attack. J Stroke Cerebrovasc Dis 2017;26:2074–81. 10.1016/j.jstrokecerebrovasdis.2017.04.012 28736132

[18] Diener HC , Cunha L , Forbes C , et al. European stroke prevention study. 2. dipyridamole and acetylsalicylic acid in the secondary prevention of stroke. J Neurol Sci 1996;143:1–13. 10.1016/S0022-510X(96)00308-5 8981292

[19] , Halkes PHA , van Gijn J , et al, ESPRIT Study Group. Aspirin plus dipyridamole versus aspirin alone after cerebral ischaemia of arterial origin (ESPRIT): randomised controlled trial. Lancet 2006;367:1665–73. 10.1016/S0140-6736(06)68734-5 16714187

